# Phase 1 Clinical Trial Evaluating the Safety and Anti-Tumor Activity of ADP-A2M10 SPEAR T-Cells in Patients With MAGE-A10+ Head and Neck, Melanoma, or Urothelial Tumors

**DOI:** 10.3389/fonc.2022.818679

**Published:** 2022-03-18

**Authors:** David S. Hong, Marcus O. Butler, Russell K. Pachynski, Ryan Sullivan, Partow Kebriaei, Sarah Boross-Harmer, Armin Ghobadi, Matthew J. Frigault, Ecaterina E. Dumbrava, Amy Sauer, Francine Brophy, Jean-Marc Navenot, Svetlana Fayngerts, Zohar Wolchinsky, Robyn Broad, Dzmitry G. Batrakou, Ruoxi Wang, Luisa M. Solis, Dzifa Yawa Duose, Joseph P. Sanderson, Andrew B. Gerry, Diane Marks, Jane Bai, Elliot Norry, Paula M. Fracasso

**Affiliations:** ^1^ Department of Investigational Cancer Therapeutics, Division of Cancer Medicine, The University of Texas MD Anderson Cancer Center, Houston, TX, United States; ^2^ Princess Margaret Hospital Cancer Centre, University of Toronto, Toronto, ON, Canada; ^3^ Division of Oncology, Department of Medicine, Washington University School of Medicine, St. Louis, MO, United States; ^4^ Massachusetts General Hospital, Harvard Medical School, Boston, MA, United States; ^5^ Adaptimmune LLC, Philadelphia, PA, United States; ^6^ Adaptimmune Limited, Abingdon, United Kingdom

**Keywords:** HNSCC, melanoma, urothelial carcinoma, ADP-A2M10, MAGE-A10, adoptive cellular therapy, TCR

## Abstract

**Background:**

ADP-A2M10 specific peptide enhanced affinity receptor (SPEAR) T-cells are genetically engineered autologous T-cells that express a high-affinity melanoma-associated antigen (MAGE)-A10-specific T-cell receptor (TCR) targeting MAGE-A10-positive tumors in the context of human leukocyte antigen (HLA)-A*02. ADP-0022-004 is a phase 1, dose-escalation trial to evaluate the safety and anti-tumor activity of ADP-A2M10 in three malignancies (https://clinicaltrials.gov: NCT02989064).

**Methods:**

Eligible patients were HLA-A*02 positive with advanced head and neck squamous cell carcinoma (HNSCC), melanoma, or urothelial carcinoma (UC) expressing MAGE-A10. Patients underwent apheresis; T-cells were isolated, transduced with a lentiviral vector containing the MAGE-A10 TCR, and expanded. Patients underwent lymphodepletion with fludarabine and cyclophosphamide prior to receiving ADP-A2M10. ADP-A2M10 was administered in two dose groups receiving 0.1×10^9^ and >1.2 to 6×10^9^ transduced cells, respectively, and an expansion group receiving 1.2 to 15×10^9^ transduced cells.

**Results:**

Ten patients (eight male and two female) with HNSCC (four), melanoma (three), and UC (three) were treated. Three patients were treated in each of the two dose groups, and four patients were treated in the expansion group. The most frequently reported adverse events grade ≥3 were leukopenia (10), lymphopenia (10), neutropenia (10), anemia (nine), and thrombocytopenia (five). Two patients reported cytokine release syndrome (one each with grade 1 and grade 3), with resolution. Best response included stable disease in four patients, progressive disease in five patients, and not evaluable in one patient. ADP-A2M10 cells were detectable in peripheral blood from patients in each dose group and the expansion group and in tumor tissues from patients in the higher dose group and the expansion group. Peak persistence was greater in patients from the higher dose group and the expansion group compared with the lower dose group.

**Conclusions:**

ADP-A2M10 has shown an acceptable safety profile with no evidence of toxicity related to off-target binding or alloreactivity in these malignancies. Persistence of ADP-A2M10 in the peripheral blood and trafficking of ADP-A2M10 into the tumor was demonstrated. Because MAGE-A10 expression frequently overlaps with MAGE-A4 expression in tumors and responses were observed in the MAGE-A4 trial (NCT03132922), this clinical program closed, and trials with SPEAR T-cells targeting the MAGE-A4 antigen are ongoing.

## Introduction

Head and neck cancer, melanoma, and urothelial carcinoma (UC) are associated with environmental carcinogens or pathogens (e.g., human papillomavirus, smoking, and sun exposure), have a high non-synonymous mutational burden, and are responsive to immunotherapeutic modalities (1). Although the overall tumor mutational burden has been associated with improved efficacy of immunotherapy, it remains unclear how specific mutational properties are associated with neoantigen presentation and response to immunotherapy (2). In addition, although there are effective treatments (including immunotherapy) for patients with these diseases, there continues to be a significant unmet medical need in managing the emergence of therapy-resistant advanced disease.

Adoptive cellular therapy is a cancer immunotherapy approach that has been studied for the treatment of hematologic and solid tumor malignancies. Primarily utilizing tumor infiltrating lymphocytes, chimeric antigen receptor T-cells, or T-cell receptor (TCR) T-cells, responses have been demonstrated with these adoptive cellular therapies in several malignancies, paving the way for approval of several chimeric antigen receptor T-cell therapies in hematologic malignancies (3–7). Although tumor-infiltrating lymphocyte therapy has shown activity in metastatic melanoma and cervical cancer (8, 9), in order for engineered T-cell therapy to be successful in the treatment of solid tumors [as has been demonstrated in human papillomavirus-associated epithelial cancers (10)], the T-cell therapy must be antigen specific to the tumor and demonstrate trafficking to and infiltration throughout the tumor.

Cancer/testis antigens are an example of tumor-specific antigens typically restricted to male germ cells in adults and are overexpressed in several cancers. Targeting cancer/testis antigens, including New York esophageal squamous cell carcinoma antigen-1 and melanoma-associated antigen (MAGE)-A4, has resulted in responses in patients with advanced synovial sarcoma, melanoma, head and neck cancer, non-small cell lung cancer, and esophagogastric cancer (11–15). MAGE-A10 is a cancer/testis protein associated with many cancers, including head and neck cancers, melanoma, and UC, and is only expressed in normal tissue in immunologically privileged sites such as testes and placenta (16–21). ADP-A2M10 is an autologous specific peptide enhanced affinity receptor (SPEAR) T-cell product genetically engineered through site-directed mutagenesis to express a TCR with increased affinity and specificity to a peptide (GLYDGMEHL) derived from MAGE-A10 complexed with human leukocyte antigen (HLA)-A*02:01 or A*02:06 (22). This SPEAR T-cell product, ADP-A2M10, was engineered because MAGE-A10 is expressed in many cancers and HLA-A*02:01 is expressed in approximately 40% of people of European descent (23). This phase 1 dose-escalation and expansion study (ADP-0022-004; https://clinicaltrials.gov: NCT02989064) assessed the safety of ADP-A2M10 for patients with advanced head and neck squamous cell carcinoma (HNSCC), melanoma, or UC.

## Materials and Methods

### Patient Eligibility and Trial Design

This multicenter, open-label, dose-escalation study included patients between 18 and 75 years of age who were HLA-A*02:01- and/or HLA-A*02:06-positive and had a histologically confirmed diagnosis of inoperable or metastatic (advanced) HNSCC, melanoma, or UC (of the bladder, ureter, urethra, or renal pelvis) that expressed MAGE-A10 (**Supplementary Methods**). Patients received at least one prior systemic therapy in the adjuvant or metastatic setting. For patients with HNSCC, a platinum-containing chemotherapy and/or immunotherapy was required for treatment in the adjuvant or metastatic setting. For those with advanced melanoma, a programmed death ligand 1 (PD-L1) inhibitor and/or a cytotoxic T-lymphocyte-associated protein-4 inhibitor were required and, in those with a *BRAF*v600 mutant melanoma, a BRAF inhibitor or a combination of BRAF and MEK inhibitors were required. Methods for HLA and MAGE-A10 testing are described in the **Supplementary Methods**. Biopsies were given both a MAGE-A10 P-score and H-score. The P-score was the MAGE-A10 immunohistochemical positivity as determined by a pathologist on the basis of both percentage of positive tumor cells and intensity of expression. The H-score was derived from the P-score by 1 × (% of 1+ cells) + 2 × (% of 2+ cells) + 3 × (% of 3+ cells). Patients had an Eastern Cooperative Oncology Group Performance Status of 0–1; adequate renal, hepatic, and hematological function; a left ventricular ejection fraction of ≥50%; and measurable disease by Response Evaluation Criteria in Solid Tumors (RECIST) v1.1 before lymphodepletion (not required before leukapheresis) (24).

**Figure 1:**
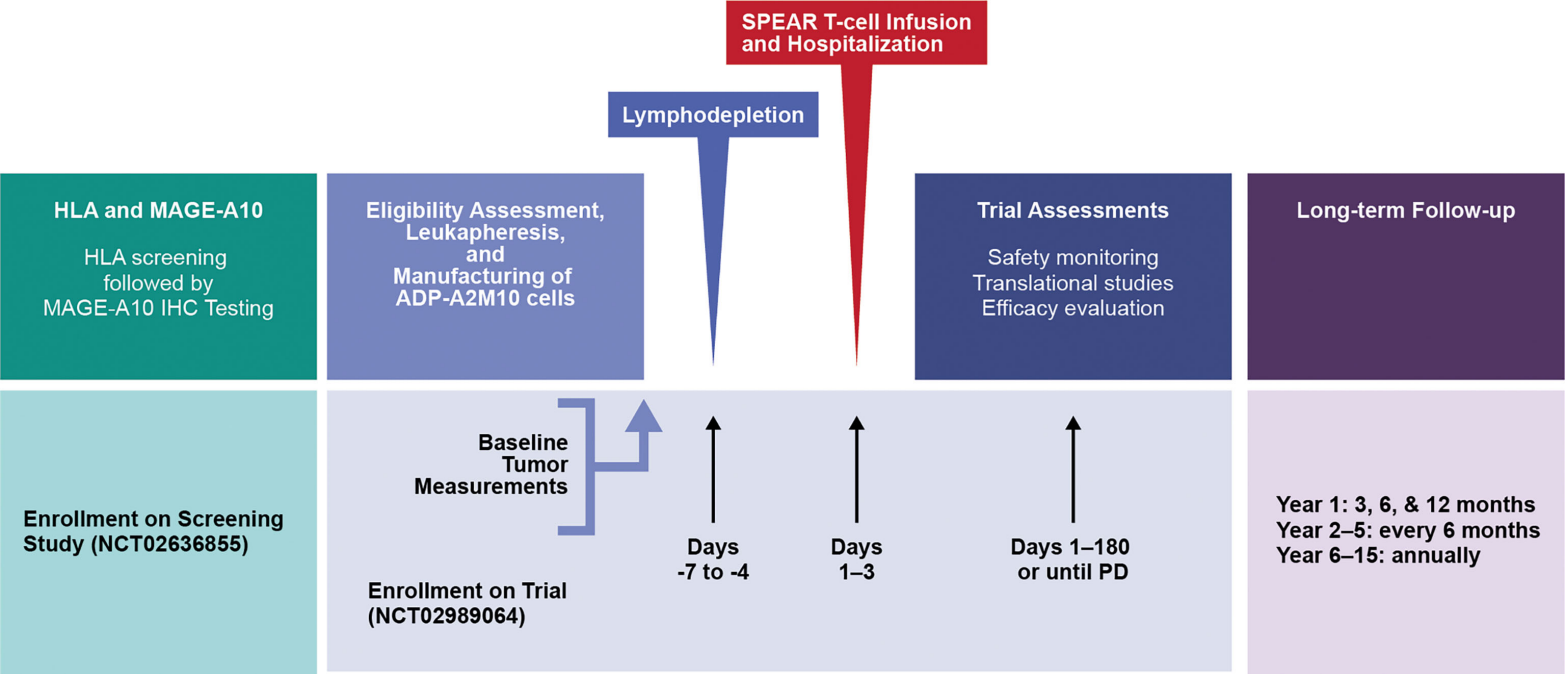
Study design. HLA, human leukocyte antigen; IHC, immunohistochemical; MAGE, melanoma-associated antigen; PD, progressive disease; SPEAR, specific peptide enhanced affinity receptor.

Eligible patients underwent leukapheresis for collection of CD3+ T-cells to manufacture ADP-A2M10 (**Supplementary Methods** and **Figure 1**). Patients were allowed treatment as “bridging therapy” during the manufacturing of their ADP-A2M10 cells and cytotoxic therapy, immunotherapy, and targeted therapy were subsequently discontinued 3, 2, and 1 weeks, respectively, from lymphodepletion. Once the ADP-A2M10 cells were available, eligibility criteria were reconfirmed, and a baseline tumor assessment was obtained. Patients then received lymphodepleting chemotherapy consisting of cyclophosphamide 600 mg/m^2^ and fludarabine 30 mg/m^2^ on days -7, -6, and -5. Given the importance of optimal lymphodepletion for effective engraftment of the ADP-A2M10 product from prior trials (25), an additional day of fludarabine 30 mg/m^2^ was incorporated on day -4 for group 3 and the expansion group (**Table 1**). Patients received the ADP-A2M10 infusion on day 1, were hospitalized for a minimum of 3 days, and were subsequently discharged at the investigator’s discretion.

**Table 1:** Dose groups – lymphodepletion and cell doses.

Dose group	Lymphodepletion regimen	Transduced cell dose (range)	Patient ID
Dose group 1	Cyclophosphamide 600 mg/m^2^/day and fludarabine 30 mg/m^2^/day on days -7, -6, and -5	0.1×10^9^ (0.08×10^9^ to 0.12×10^9^)	1–3
Dose group 2^a^	Cyclophosphamide 600 mg/m^2^/day and fludarabine 30 mg/m^2^/day on days -7, -6, and -5	1×10^9^ (0.5×10^9^ to 1.2×10^9^)	–
Dose group 3	Cyclophosphamide 600 mg/m^2^/day on days -7, -6, and -5 and fludarabine 30 mg/m^2^/day on days -7, -6, -5, and -4	5×10^9^ (>1.2×10^9^ to 6×10^9^)	4–6
Expansion group	Cyclophosphamide 600 mg/m^2^/day on days -7, -6, and -5 and fludarabine 30 mg/m^2^/day on days -7, -6, -5, and -4	5×10^9^ (1.2×10^9^ to 15×10^9^)	7–10

a No patients were treated in dose group 2 as this dose group was removed from the protocol based on the Safety Review Committee assessment of safety data from patients in an identical dose group with the same investigational agent in Study ADP-0022-003 (NCT02592577).

As described in **Table 1**, ADP-A2M10 dose ranges were: group 1: 0.08 × 10^9^ - 0.12 × 10^9^ cells; group 2: 0.5 × 10^9^ - 1.2 × 10^9^ cells; and group 3: >1.2 × 10^9^ - 6.0 × 10^9^ cells. Intervals of 21 days (dose group 1) and 7 days (dose group 2 and dose group 3) between dosing each patient were included to evaluate safety. No patients were treated in dose group 2 because the group was removed from the protocol based on the Safety Review Committee assessment of safety data from patients in an identical dose group with the same investigational agent in Study ADP-0022-003 (NCT02592577). Therefore, ADP-A2M10 was escalated directly from group 1 to group 3. Following the evaluation of safety for group 3, an expansion group of up to 15 patients was dosed at 1.2 × 10^9^ - 15 × 10^9^ ADP-A2M10. The interventional phase lasted until progressive disease (PD), after which patients were rolled over into long-term follow-up for up to 15 years post-infusion or until death or withdrawal.

Tumor biopsies were required at screening and optional at baseline and after T-cell infusion. Screening biopsies were either archival tumor samples or fresh samples required for eligibility. Baseline biopsies were collected from 2 months to 1 week prior to lymphodepletion, and post-infusion biopsies were collected from week 3 to 12 after infusion.

The protocol and amendments were approved by the institutional review board or ethics committee at each participating site, and the trial was conducted in accordance with the International Council for Harmonisation Good Clinical Practice Guideline and the principles of the Declaration of Helsinki. All patients provided written informed consent prior to enrollment as per institutional guidelines. No patients or members of the public were involved in the design of this clinical trial.

### Assessment of Toxicities and Responses

Safety and tolerability assessments were conducted at each study visit as follows: baseline; day -7 to -3, 1–5, and 8; week 2, 3, 4, 5, 6, 8, 10, 12, 18, and 24; every 3 months until year 2; every 6 months from year 2 to 5, or until disease progression; and throughout long-term follow-up for up to 15 years. Adverse events (AEs) of special interest were cytokine release syndrome (CRS), prolonged cytopenia, neurotoxicity, and graft versus host disease. AEs were graded in accordance with the National Cancer Institute Common Terminology Criteria for Adverse Events v4.0, with two exceptions. CRS was graded as described previously (26), and prolonged cytopenias were graded as grade ≥3 neutropenia, anemia, or thrombocytopenia in accordance with Common Terminology Criteria for Adverse Events v5.0 and persisting for ≥4 weeks from ADP-A2M10 treatment.

A dose-limiting toxicity (DLT) was defined as a grade ≥3 AE within the first 30 days after administration of ADP-A2M10, regardless of the investigator’s assessment of relationship to ADP-A2M10. In evaluating potential DLTs, grade 3 or 4 CRS resolving to grade ≤2 within 7 days and toxicities of any grade considered attributable to the underlying malignancy, lymphodepletion chemotherapy, or otherwise clearly unrelated to the ADP-A2M10 were deemed not a DLT by the Safety Review Committee.

Patients were followed for 15 years after treatment with genetically modified T-cells in accordance with US Food and Drug Administration and European Medicines Agency guidance (27–29) to identify potential gene therapy-related delayed AEs, for molecular replication competent lentivirus testing (quantitative PCR for the vesicular stomatitis virus – G protein DNA sequence), and — if necessary — for insertional oncogenesis (**Supplementary Methods**).

Efficacy was evaluated using RECIST v1.1 at the following study visits: baseline (within 7 days of lymphodepleting chemotherapy); weeks 6, 12, 18, and 24, then every 3 months until year 2; and every 6 months from year 2 to 5, or until disease progression; and at completion. Although the evaluation of response was done from the day of lymphodepletion, the duration of response was assessed from day of ADP-A2M10 infusion (7 days after lymphodepletion).

### Translational Studies

Translational studies included the assessment of ADP-A2M10 T-cell persistence in peripheral blood and bone marrow, if indicated; serum cytokine levels during CRS events; transcriptomic analyses for antigen processing machinery, CD3+ T-cells, and CD8+ T-cells; T-cell exhaustion evaluations in pre-infusion tumor samples; presence of ADP-A2M10 T-cells in post-infusion tumor samples; and PD-L1 expression in pre- and post-infusion tumor samples.

Persistence of transduced ADP-A2M10 was assessed at BioAgilytix (Boston, Massachusetts) by measuring the number of copies of integrated lentiviral vectors (Psi element sequence) per microgram of genomic DNA from peripheral blood, when applicable, in bone marrow mononuclear cells by quantitative PCR at the following time points: baseline; day 2, 4, and 8; week 2, 4, 6, 12, and 24; and every 3 months until year 2.

Levels of serum cytokines were collected at baseline; day 1–5, and 8; week 2, 3, 4, 6, 12 and 24; and every 3 months and were evaluated as previously described (25).

Transcriptomic analyses were performed at CellCarta (Antwerp, Belgium) or the University of Texas MD Anderson Cancer Center (Houston, Texas) as previously reported (25), with modifications described in the **Supplementary Methods**. The following gene signatures were used for analyses: antigen processing machinery (30), CD3+ T-cells, CD8+ T-cells (31) (NanoString Technologies, Seattle, Washington), and T-cell exhaustion (32).

RNA *in situ* hybridization for ADP-A2M10 TCR was performed on the Ventana Discovery Ultra automation platform (Roche Diagnostics, Indianapolis, Indiana) using the RNAscope 2.5 LS Red kit (Advanced Cell Diagnostics, Newark, California) and RNAscope probes specific to ADP-A2M10 TCR (Advanced Cell Diagnostics) according to the manufacturer’s instructions. RNA *in situ* hybridization assay was followed by CD3 chromogenic precipitate immunohistochemistry (anti-CD3 (2GV6), rabbit monoclonal primary antibody, Roche Diagnostics, Indianapolis, Indiana) using the DISCOVERY Teal horseradish peroxidase detection kit (Roche Diagnostics) (**Supplementary Methods**).

PD-L1 expression was determined at a Clinical Laboratory Improvement Amendments-certified and Belgian Accreditation Organization and College of American Pathologists-accredited laboratory (CellCarta, Antwerp, Belgium) or the University of Texas MD Anderson Cancer Center (Houston, Texas) using PD-L1 IHC 22C3 pharmDx (Agilent, Santa Clara, California). Tumor Proportion Score and Combined Positive Score for PD-L1 expression were assessed as recommended by the manufacturer.

### Statistical Considerations

The primary objective was evaluation of the safety and tolerability of ADP-A2M10. Secondary objectives were assessment of anti-tumor activity according to RECIST v1.1 and evaluation of potential gene therapy-related delayed AEs. Exploratory objectives were to assess the persistence of transduced ADP-A2M10 in peripheral blood and their trafficking into tumor tissue, the production of cytokines during CRS events, and the tumor and tumor microenvironment characteristics.

Progression-free survival, overall survival, and duration of stable disease (SD) were summarized and graphically displayed using the Kaplan-Meier method to estimate the 25th, 50th (median), and 75th percentiles with corresponding 95% CIs. The intent-to-treat population for analysis was defined as all eligible patients who were enrolled in the study. The modified intent-to-treat population included all patients in the intent-to-treat population who received at least one ADP-A2M10 infusion. The modified intent-to-treat population was the primary population for the safety and efficacy analyses.

## Results

### Patient Characteristics

From September 2017 to September 2019, 10 patients underwent lymphodepletion and were treated with ADP-A2M10 (**Supplementary Results**, **Supplementary Table 1**, and **Supplementary Figure 1**). Of the 10 treated patients, patients 1–3, patients 4–6, and patients 7–10 were treated in dose group 1, dose group 3, and the expansion group, respectively. Demographic and baseline characteristics are detailed in **Table 2**. The median age of the treated patients was 63 years (range: 46–76 years), race included nine white and one other, and ethnicity included nine non-Hispanic or Latino and only one Hispanic or Latino. Four patients (patients 1, 2, 5, and 7) had HNSCC, three patients (patients 3, 8, and 9) had cutaneous melanoma, and three patients (patients 4, 6, and 10) had UC. HLA and MAGE-A10 expression, and ADP-A2M10 cell dose are as described. All 10 treated patients had at least one prior systemic therapy (median: two; range: two to four). Nine reported prior chemotherapy, nine reported prior immunotherapy, two reported prior targeted therapy, and eight reported prior radiotherapy.

**Table 2:** HLA and MAGE-A10 expression, ADP-A2M10 dose, and response in individual patients (mITT) with various tumor types at screening.

Patient ID	Age (years) Sex	Tumor Type	Prior Therapy^a^	HLA-A Allele 1/Allele 2	MAGE-A10 P-score^b^ (% <1+, 1+, 2+, 3+)	MAGE-A10 H-score^c^	Actual ADP-A2M10 dose (cells ×10^9^)	Response/(Max %Change in SLD) DoSD^d^
1	66 M	HNSCC	1, 2, 3, 4	01:01 02.01	0, 10, 30, 60	250	0.1	PD
2	61 M	HNSCC	1, 2, 4	02:01 3:01	30, 20, 40, 10	130	0.1	NE
3	47 M	Mel	1, 2, 4	02:01 25:01	0, 40, 30, 30	190	0.09	PD
4	58 M	UC	1, 2, 4	02:01 26:01	35, 40, 20, 5	95	5.26	SD (3.8%) 65 days
5	46 F	HNSCC	1, 2, 4	02:0124:AUJRX	5, 5, 70, 20	205	5.99	PD
6	66 M	UC	1, 3	02:01 26:01	20, 30, 30, 20	150	5.51	SD (12.3%) 246 days
7	76 M	HNSCC	1, 2, 4	02:01 11:01	0, 0, 20, 80	280	3.97	PD
8	47 F	Mel	1, 2	01:01 02:01	60, 5, 5, 30	105	4.87	PD
9	65 M	Mel	2, 4	01:01 02:01	10, 20, 50, 20	180	6.53	SD (1.1%) 122 days
10	68 M	UC	1, 2, 4	02:01 25:01	0, 5, 5, 90	285	13.63	SD (0%) 36 days

a 1, Chemotherapy; 2, Immunotherapy; 3, Targeted therapy; 4, Radiotherapy.

b P-score is immunohistochemical positivity determined by a pathologist based on both percentage of positive tumor cells and intensity of expression.

c H-score is derived by 1 × (% of 1+ cells) + 2 × (% of 2+ cells) + 3 × (% of 3+ cells).

d DoSD only analyzed in patients with SD.

DoSD, duration of stable disease; F, female; HLA, human leukocyte antigen; HNSCC, head and neck squamous cell carcinoma; M, male; MAGE, melanoma-associated antigen; Mel, melanoma; mITT, modified intent-to-treat; NE, not evaluable; PD, progressive disease; SD, stable disease; SLD, sum of the longest diameters of the target lesions; UC, urothelial carcinoma.

### Treatment and AEs

Of the 10 treated patients, three in dose group 1, three in dose group 3, and four in the expansion group were treated with ADP-A2M10 within the dose range of 0.09 to 13.6×10^9^ transduced cells (**Table 2**). There were no DLTs during the dose-escalation portion of the study and there were no apparent differences in AE grades across the cell doses of ADP-A2M10 in the two dose groups and the expansion group.

All 10 treated patients experienced at least one AE. AEs of grade ≥3 occurring in ≥20% of patients are shown in **Table 3**. The most common AEs reported were leukopenia/white blood cells decreased, lymphopenia/lymphocyte count decreased, and neutropenia/neutrophil count decreased, which occurred in all 10 patients. There were no grade 5 AEs.

**Table 3:** AEs in ≥20% of all patients of any grade and all patients of grade ≥3: mITT population.

AEs by Preferred Term	Number of patients (N = 10)		
	Any grade	Grade 3	Grade 4
Leukopenia/white blood cell count decreased	10	0	10
Lymphopenia/lymphocyte count decreased	10	0	10
Neutropenia/neutrophil count decreased	10	0	10
Anemia/red blood cell count decreased	9	9	0
Thrombocytopenia/platelet count decreased	6	1	4
Hyponatremia	6	5	0
Hypophosphatemia	4	1	1
Fatigue	7	2	0
Tumor pain	3	2	0
Rash	5	0	1
Pyrexia	6	1	0
Decreased appetite	5	1	0
Constipation	4	1	0
Hypotension	4	1	0
Hypocalcemia	3	1	0
Abdominal pain	2	1	0
Acute kidney injury	2	1	0
CRS	2	1	0
Dyspnea	2	1	0

AE, adverse event; CRS, cytokine release syndrome; mITT, modified intent-to-treat.

Nine patients had AEs definitely, probably, or possibly related to ADP-A2M10 therapy (**Supplementary Table 2**). The most common related AEs were pyrexia, rash, and cytopenias. Two patients (patients 9 and 10, expansion group) reported serious AEs that were related to ADP-A2M10. Patient 9 developed grade 3 CRS, which resolved with treatment as described below. The other (patient 10) developed several events, including grade 1 acute kidney injury and a grade 2 maculo-papular rash, which both resolved, and pancytopenia, which was resolving at the time of the patient’s death of PD as described below.

Of the 10 patients treated, two entered long-term follow-up; however, they subsequently died due to PD. No AEs related to gene-modified cell therapy were reported, all samples tested for replication competent lentivirus were negative, and no patients met the criteria for assessment of insertional oncogenesis.

#### AEs of Special Interest

CRS was reported in two of the 10 treated patients (patients 7 and 9, expansion group) with severity of grade 1 and 3, respectively, occurring on day 1 and 7, respectively, after ADP-A2M10 infusion, and lasting only 1 day. Although patient 7 with HNSCC received no treatment, patient 9 with melanoma and grade 3 CRS received tocilizumab and methylprednisolone. A transient increase in serum cytokines, including interferon γ, interleukin (IL)-6, IL-8, and IL-10, was observed after ADP-A2M10 T-cell infusion in all patients. There was no marked difference in serum cytokines (i.e., interferon γ, IL-6, IL-8) between those who had CRS and those who did not have CRS (**Supplementary Figure 2**); however, the small sample limits the conclusions that can be drawn.

Three patients experienced prolonged cytopenia. The first patient (patient 2, dose group 1) was noted to have pancytopenia during week 2, which was treated with filgrastim and was ongoing at the time of the patient’s death from PD during week 7. The second patient (patient 4, dose group 3) had a history of grade 2 anemia at baseline, which changed to grade 3 anemia following lymphodepletion and resolved to grade 2 on week 4. However, from week 5 until week 11 of the interventional phase, the patient had intermittent grade 3 anemia possibly related to the patient’s prior lymphodepletion and subsequent comorbidities. The third patient (patient 10, expansion group) was noted to have pancytopenia on week 3, thought to be related to lymphodepletion and ADP-A2M10. The patient underwent a bone marrow biopsy, was found to have a hypocellular marrow with panhypoplasia, and was treated with filgrastim/peg-filgrastim and eltrombopag. With cell counts demonstrating a normal white blood cell and neutrophil count and grade 2 platelets and hemoglobin, the patient died of PD in week 6. There was no evidence that ADP-A2M10 cells were enriched in bone marrow compared with peripheral blood (**Supplementary Figure 3**).

Two patients (patients 9 and 8, expansion group) had neurotoxicity, grade 1 intermittent somnolence, and grade 1 concentration impairment, respectively, possibly related to ADP-A2M10. The patient with grade 1 intermittent somnolence had this symptom associated with grade 3 CRS, which resolved in 1 day. Patient 8 with grade 1 concentration impairment was noted to have this symptom beginning 6 days after ADP-A2M10 and was ongoing at week 6 when the patient was noted to have PD. There were no graft versus host disease events reported.

### Response Data

Nine of the 10 treated patients were evaluable for response by RECIST v1.1 (**Table 2**). One patient (patient 2 with HNSCC) progressed and died prior to evaluation. Four patients (patients 4, 6, and 10 with UC and patient 9 with melanoma) had SD and five patients (three with HNSCC and two with melanoma) had PD. Of the patients with SD, two patients were in dose group 3, and two patients were in the expansion group.

The median progression-free survival for all patients was 46.5 days (range: 18–246 days), with median survival for dose group 3 at 65 days (range: 18–246 days) and for the expansion group at 46.5 days (range: 31–122 days). The median overall survival for all patients was 171 days (range: 44–319 days), with median survival for dose group 3 at 106 days (range: 59–250 days) and for the expansion group at 236 days (range: 44–319 days). The Kaplan-Meier estimate of median duration of SD was 122 days (range: 36-246 days).

Of the 10 patients treated, one died prior to evaluation due to PD while in the interventional phase. The remaining nine patients were evaluable and ended the interventional phase due to PD. Of these nine patients, three died due to PD prior to entering long-term follow-up, three did not consent to long-term follow-up, one withdrew consent prior to entering long-term follow-up, and two entered long-term follow-up and subsequently died due to PD.

### Translational Studies

**Figure 2:**
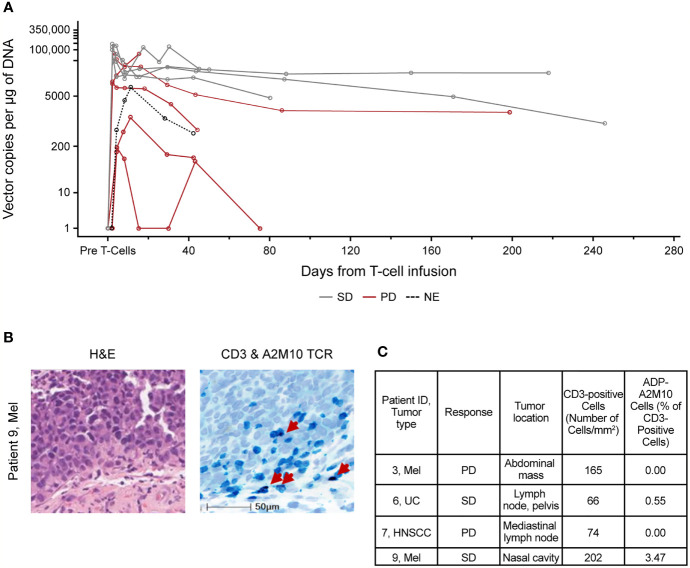
ADP-A2M10 were detected in peripheral blood and tumor tissue after infusion. **(A)** Persistence of ADP-A2M10 was measured by quantitative PCR of the Psi element sequence in genomic DNA extracted from peripheral blood mononuclear cells. Data points are colored by response. **(B)** Representative fields for detection of CD3+ and/or ADP-A2M10 TCR+ cells by CD3 immunohistochemical/RNA *in situ* hybridization assay in tumor tissue of patient 9 collected within 12 weeks after infusion. In the right image, CD3+ cells are shown in teal, ADP-A2M10 TCR+ cells are shown in purple, and nuclei are shown in light blue (hematoxylin stain). **(C)** Result table for CD3 immunohistochemical/RNA *in situ* hybridization duplex assays reporting the detection of ADP-A2M10 in two of four post-infusion tumor samples collected from the study patients. H&E, hematoxylin and eosin stain; HNSCC, head and neck squamous cell carcinoma; Mel, melanoma; NE, not evaluable; PD, progressive disease; SD, stable disease; TCR, T-cell receptor; UC, urothelial carcinoma.

The persistence kinetics for ADP-A2M10-transduced cells varied among patients. However, the presence of the transduced cells was observed in all patients throughout the follow-up period and up to 9 months post-infusion (**Figure 2A**). On average, peak persistence was higher in patients from dose group 3 and expansion group compared with patients from dose group 1 (**Supplementary Table 3**). Time to peak persistence was comparable in patients across the groups (**Supplementary Table 3**). Peak persistence trended higher in patients with SD; however, the small sample limits the ability to draw conclusions (**Figure 2A**).

ADP-A2M10 infiltration was examined in four biopsies collected within 12 weeks after infusion. ADP-A2M10 was observed in tumor samples of two SD patients treated in dose group 3 and the expansion group: patient 6 with UC and patient 9 with melanoma (**Figures 2B, C**). T-cell infiltration was not detected in the tumor tissue of two patients with PD: patient 3 with melanoma from dose group 1 and patient 7 with HNSCC from the expansion group (**Figure 2C**).

**Figure 3:**
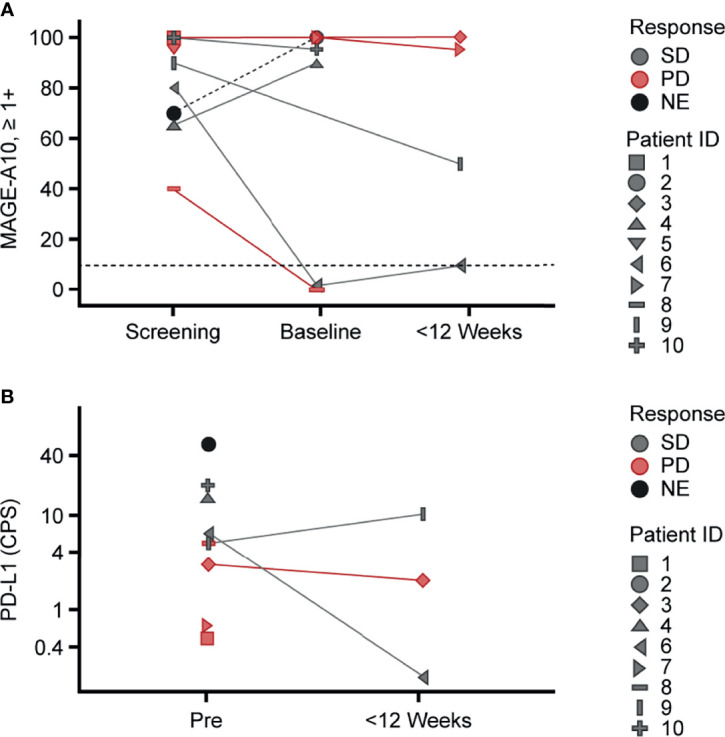
Variability of MAGE-A10 and PD-L1 expression in tumor tissue across the study patients. Pre-infusion (screening and baseline) and post-infusion biopsies collected within 12 weeks after infusion were used for **(A)** MAGE-A10 expression and **(B)** PD-L1 expression evaluation. **(A)** MAGE-A10 expression was assessed by MAGE-A10 immunohistochemical staining and plotted as percentage of tumor cells with 1+, 2+ and 3+ intensities. Horizontal lines designate the cut-off of 10% of tumor with ≥1+ intensity of staining. **(B)** PD-L1 expression was assessed using PD-L1 IHC 22C3 pharmDx assay and plotted in terms of CPS. **(A, B)** Data points are colored by response. Patient IDs are indicated by shape. CPS, Combined Positive Score; MAGE, melanoma-associated antigen; NE, not evaluable; PD, progressive disease; PD-L1, programmed death ligand 1; Pre, pre-infusion; SD, stable disease.

Among seven patients treated in both dose group 3 and the expansion group, three patients with UC and one patient with melanoma had SD, whereas two patients with HNSCC and one patient with melanoma had PD. To investigate the lack of clinical responses across indications, MAGE-A10 protein expression in tumor cells pre- and post-infusion, PD-L1 protein expression in tumor and stromal cells pre- and post-infusion, CD3+ T-cell, CD8+ T-cell, T-cell exhaustion, and antigen processing machinery gene expression pre-infusion were evaluated in accordance with tumor sample availability. The characteristics measured showed variability across patients and did not significantly differ between indications and response (**Figure 3** and **Supplementary Figure 4**).

Interestingly, there were differences in MAGE-A10 expression in the screening biopsies of patients 6 (SD) and 8 (PD) collected 12 and 7 months before infusion, respectively, and sequential baseline biopsies taken within 3 weeks before infusion. Both baseline biopsies were negative for MAGE-A10 expression based on the study cut-off (**Figure 3A**). The screening biopsies were collected during resections whereas the baseline biopsies were core biopsies. Given the variation in size, these differences in MAGE-A10 levels may be explained by inter- and intra-lesional tumor heterogeneity reported for UC and melanoma (33, 34). Four and three post-infusion biopsies were available for MAGE-A10 and PD-L1 expression evaluation, respectively. There were no significant effects of ADP-A2M10 cell infusion on the expression of these proteins (**Figures 3A, B**). With limited patient data, there was no evidence indicating that any of the selected pre- and post-infusion tumor characteristics dictated the response to the ADP-A2M10 therapy.

## Discussion

ADP-0022-004 is a single-arm, open-label, phase 1, dose-escalation study to assess ADP-A2M10 in advanced HNSCC, melanoma, and UC. Ten patients were treated with lymphodepleting chemotherapy followed by ADP-A2M10. Dose escalation, administering a target of 5×10^9^ ADP-A2M10, was safely completed, and the expansion group commenced (up to 15 × 10^9^ ADP-A2M10). There were no DLTs in the dose-escalation phase. ADP-A2M10 with lymphodepleting chemotherapy consisting of cyclophosphamide 600 mg/m^2^ for 3 days and fludarabine 30 mg/m^2^ for 3 or 4 days was associated with an acceptable safety profile in this study. Cytopenias were the most common AEs reported. Two patients had serious AEs related to therapy (CRS in one patient; pancytopenia, maculo-papular rash, and acute kidney injury in the other). Six patients reported seven treatment-related AEs of special interest: prolonged cytopenia (three events), CRS (two events, grades 1 and 3), and AEs consistent with grade 1 neurotoxicity (two events).

Although there were four patients with SD, no objective responses other than SD were reported. Factors that may contribute to the efficacy of autologous TCR-engineered T-cells include antigen expression, T-cell dose, TCR expression levels, tumor trafficking and persistence in peripheral blood, and immunosuppression (3, 4, 7). High expression of tumor-specific antigens as designated by H-score has been associated with responses in patients with synovial sarcoma (15, 25). Although the MAGE-A10 H-score was ≥100 in most patients’ tumors, there were no patients with partial response and no obvious differences in MAGE-A10 expression in patients with SD or PD. Furthermore, two patients had screening biopsies collected over 6 months before ADP-A2M10 infusions as well as baseline biopsies taken within a 3-week period before ADP-A2M10 infusion. The screening biopsies were collected during resections whereas the baseline biopsies were core biopsies. While both screening biopsies were positive for MAGE-A10, the baseline biopsies were not. It is unclear whether these differences were related to changes in expression levels with subsequent treatment or variation in size of the biopsies. In addition, inter- and intra-lesional tumor heterogeneity has been reported for UC and melanoma (33, 34). It is possible that this heterogeneity may also contribute to the lack of response even in patients with a high H-score. Nonetheless, larger tumor biopsies closer to the time of treatment would be important in future studies.

While antigen expression may be required, it alone is not sufficient to result in a response without other contributing factors such as cell dose and T-cell infiltration into the tumor microenvironment. Anti-tumor responses have been more often observed in those patients who received New York esophageal squamous cell carcinoma antigen-1 transduced cell doses of ≥1×10^9^ (11) and doses of ≥5×10^9^ afamitresgene autoleucel (ADP-A2M4) (15). Seven patients received a dose of ≥5×10^9^ ADP-A2M10. Interestingly, SD was only demonstrated in those patients. Additionally, four patients had post-treatment tumor biopsies collected; and, importantly, ADP-A2M10 trafficking into the tumor was observed in two patients, both with SD and with ADP-A2M10 cell doses >5×10^9^. Moreover, peak persistence trended higher in patients from dose group 3 and the expansion group, and in those with SD. Although the limited number of patients with HNSCC, melanoma, and UC treated in this study does not allow any firm conclusions to be drawn, there appears to be a correlation toward a higher ADP-A2M10 cell dose of 5×10^9^ and ADP-A2M10 trafficking into the tumor, persistence, and SD. Finally, the efficacy of ADP-A2M10 therapy could be limited by pre-existing and acquired intratumoral immunosuppression. Novel combinations of SPEAR T-cell monotherapy with approved drugs, such as checkpoint inhibitors, or development of next−generation SPEAR T-cells co-expressing additional immunoregulatory molecules, such as CD8α or IL-7/chemokine (C-C motif) ligand 19, may increase anti-tumor activity by overcoming immunosuppression and improving clinical responses in patients with epithelial cancers (12, 35).

In this study, ADP-A2M10, an innovative autologous SPEAR T-cell manufactured product, was successfully administered to patients with several different solid tumor malignancies expressing MAGE-A10. An acceptable safety profile with no evidence of toxicity related to off-target binding or alloreactivity was demonstrated. Trafficking of ADP-A2M10 cells in the blood and to the tumor was confirmed and peak persistence was greater in patients from the higher dose groups compared with the lower dose group. Four of the 11 patients had clinical benefit with SD. Nonetheless, among HLA-eligible patients with HNSCC, melanoma, or UC, the frequency of positivity for the MAGE-A10 antigen was lower than expected, making enrollment challenging. While this trial was in progress, Study ADP-0044-001 (NCT03132922) targeting the MAGE-A4 antigen with ADP-A2M4 commenced and significant responses were seen in patients with non-small cell lung cancer, head and neck cancer, and synovial sarcoma (13). Given data that MAGE−A10 expression frequently overlaps with MAGE-A4 expression (Adaptimmune, data on file), this trial was closed and several trials with adoptive cellular therapies targeting MAGE-A4, including a registrational study in sarcoma, are now ongoing (NCT03132922, NCT04044768, and NCT04044859). Lastly, the findings from this study suggest that targeting MAGE-A10 with this TCR could be further optimized through improvements in T-cell manufacture, enhancement of the phenotypic composition of the T-cell product, or by targeting multiple antigens such as MAGE-A4 and A10 that are co-expressed in some tumors (as has been recently explored in lymphoma (36)). Translational analyses, including phenotyping of the patient’s manufactured product and post-infusion T-cells for markers of immune activation and/or exhaustion, may help the field’s understanding of what may be a desired phenotype for future T-cell products for mono- or multi-antigen targeting T-cells. Furthermore, these analyses will also contribute to understanding which patients may respond to adoptive T-cell therapy with engineered affinity-enhanced TCRs.

## Author’s Note

Data from this manuscript were presented at the Society of Immunotherapy of Cancer 2020 Meeting; November 11-14, 2020; Journal for ImmunoTherapy of Cancer. Dec 2020, 8 (Suppl 3) A174; doi: 10.1136/jitc- 2020-SITC2020.0285.

## Data Availability Statement

All data relevant to the study are included in the article or uploaded as supplementary information. The raw data sets generated, used, and analyzed during the current study are available from the corresponding author on reasonable request. The transcriptomic sequencing data presented in this study can be found in an online repository (Gene Expression Omnibus, https://www.ncbi.nlm.nih.gov/geo/; accession number: GSE195530).

## Ethics Statement

The protocol and amendments were approved by the institutional review board or ethics committee at each participating site, and the trial was conducted in accordance with the International Council for Harmonisation Good Clinical Practice Guideline and the principles of the Declaration of Helsinki. All patients provided written informed consent prior to enrollment as per institutional guidelines

## Author Contributions

All authors were involved in the conception or design of the work or the acquisition, analysis, and interpretation of data for the work; drafted the work or revised it critically for important intellectual content; provided final approval of the version to be published; and agree to be accountable for all aspects of the work by ensuring that questions related to the accuracy or integrity of any part of the work are appropriately investigated and resolved. AS: coordinated/oversaw study management. DD: provided technical contributions for the NanoString assay. LS: designed/carried out the translational experiments. SF: designed the translational studies. ZW: designed/carried out the translational experiments.

## Funding

This study received funding from Adaptimmune Ltd.

## Conflict of Interest

ABG: holds stock options in Adaptimmune. AS, DB, DM, EN, FB, JB, J-MN, JS, RB, RW, SF, and ZW: employees of Adaptimmune. DH: research/ grant funding from AbbVie, Adaptimmune, Aldi-Norte, Amgen, AstraZeneca, Bayer, BMS, Daiichi-Sankyo, Deciphera, Eisai, Erasca, Fate Therapeutics, Genentech, Genmab, Infinity, Kite, Kyowa, Lilly, LOXO, MedImmune, Merck, Mirati, Mologen, Navier, NCI-CTEP, Novartis, Numab, Pfizer, Pyramid Bio, SeaGen, Takeda, Turning Point Therapeutics, Verastem, and VM Oncology; compensation for travel, accommodations, and expenses from AACR, ASCO, Bayer, Genmab, SITC, and Telperian; consulting, speaker, or advisory roles for Acuta, Adaptimmune, Alkermes, Alpha Insights, Amgen, Atheneum, AUM Biosciences, Axiom, Barclays, Baxter, Bayer, Boxer Capital, BridgeBio, CDR-life AG, COG, COR2ed, Ecor1, Genentech, Gilead, GLG, Group H, Guidepoint, HCW Precision, Immunogen, Infinity, Janssen, Liberium, Medscape, Numab, Oncologia Brasil, Pfizer, Pharma Intelligence, POET Congress, Prime Oncology, Seattle Genetics, ST Cube, Takeda, Tavistock, Trieza Therapeutics, Turning Point, WebMD, and Ziopharm; other ownership interests in OncoResponse (Founder) and Telperian Inc (Advisor).

ED: grant/research support from Aileron Therapeutics, Amgen, Aprea Therapeutics, Astex Pharmaceuticals, Bayer HealthCare Pharmaceuticals Inc., Bellicum Pharmaceuticals, BOLT Therapeutics, Compugen Ltd, Immunocore LTD, Immunomedics, Mereo BioPharma 5 Inc, NCI, PMV Pharma, Sanofi, SeaGen, TRACON Pharmaceuticals Inc., Triumvira, and Unum Therapeutics; advisory board for BOLT Therapeutics. MB: grant/contract funding for investigator-initiated clinical trials from Merck and Takara Bio; advisory boards for Adaptimmune, BMS, EMD Serono, GlaxoSmithKline, Immunocore, Instil Bio, Iovance, Merck, Novartis, Pfizer, and Sanofi; has attended speaking engagements for BMS, Merck, Novartis, and Pfizer; Safety Review Committees for Adaptimmune and GlaxoSmithKline.

MF: consultant for Arcellx, BMS, Iovance, Kite, and Novartis. PF: employee of Adaptimmune; holds stock in Adaptimmune and Bristol-Myers Squibb; has received compensation for travel and congress meetings. RS: research support from Amgen and Merck; has received fees as a consultant or SAB member from Array Biopharma, Asana Biosciences, AstraZeneca, BMS, Eisai, Iovance, Merck, Novartis, OncoSec, Pfizer, and Replimune. RP: grant funding from Janssen; personal fees from AstraZeneca, Bayer, BMS, Dendreon, EMD, Genentech/ Roche, Genomic Health, Jounce Therapeutics, Merck, Sanofi, and Serono/Pfizer; nonfinancial support from BMS and Genentech/Roche.

The remaining authors declare that the research was conducted in the absence of any commercial or financial relationships that could be construed as a potential conflict of interest.

The authors declare that this study received funding from Adaptimmune Ltd. The funder had the following involvement with the study: study design and analysis and interpretation of the data.

## Publisher’s Note

All claims expressed in this article are solely those of the authors and do not necessarily represent those of their affiliated organizations, or those of the publisher, the editors and the reviewers. Any product that may be evaluated in this article, or claim that may be made by its manufacturer, is not guaranteed or endorsed by the publisher.

## Abbreviations

ADP-A2M4, afamitresgene autoleucel; ADP-A2M10, ADP-A2M10 SPEAR T-cells; AE, adverse event; CRS, cytokine release syndrome; DLT, dose-limiting toxicity; HLA, human leukocyte antigen; HNSCC, head and neck squamous cell carcinoma; IL, interleukin; MAGE, melanoma-associated antigen; PD, progressive disease; PD-L1, programmed death ligand 1; RECIST, Response Evaluation Criteria in Solid Tumors; SD, stable disease; SPEAR, specific peptide enhanced affinity receptor; TCR, T-cell receptor; UC, urothelial carcinoma.
