# Supplementary material for: Phase 1 Clinical Trial Evaluating the Safety and Anti-Tumor Activity of ADP-A2M10 SPEAR T-Cells in Patients With MAGE-A10+ Head and Neck, Melanoma, or Urothelial Tumors
Source: Front Oncol. 2022 Mar 18;12:818679. doi: 10.3389/fonc.2022.818679 (PMC8972123; doi:10.3389/fonc.2022.818679)
Supplement: Supplementary file 1 [file DataSheet_1.docx]

**SUPPLEMENTARY APPENDIX**

**TABLE OF CONTENTS**

[SUPPLEMENTARY METHODS 3](#_Toc87612710)

[HLA and MAGE-A10 Testing 3](#_Toc87612711)

[T-cell Manufacturing 3](#_Toc87612712)

[Gene Therapy-Related Delayed Adverse Events, Molecular Replication Competent Lentivirus Testing, and Insertional Oncogenesis 3](#_Toc87612713)

[Translational 4](#_Toc87612714)

[Transcriptomic Analysis 4](#_Toc87612715)

[RNA In Situ Hybridization 4](#_Toc87612716)

[SUPPLEMENTARY RESULTS 4](#_Toc87612717)

[Patient Characteristics 4](#_Toc87612718)

[SUPPLEMENTARY TABLES 6](#_Toc87612719)

[Supplementary Table 1. ADP-0022-004 patients screened for HLA and MAGE-A10 under screening protocol ADP-0000-001. 6](#_Toc87612720)

[Supplementary Table 2. All AEs related^a^ to ADP-A2M10 by grade ≥3 (mITT population). 7](#_Toc87612721)

[Supplementary Table 3. Peak persistence and time to peak persistence by dose group (mITT population). 8](#_Toc87612722)

[SUPPLEMENTARY FIGURES 9](#_Toc87612723)

[Supplementary Figure 1. Patient disposition. 9](#_Toc87612724)

[Supplementary Figure 2. Elevation of selected serum cytokines after infusion in mITT population. 10](#_Toc87612725)

[Supplementary Figure 3. There was no evidence of enrichment of ADP-A2M10 in bone marrow of patient 10 compared with peripheral blood. 11](#_Toc87612726)

[Supplementary Figure 4. Variability of APM, CD3+ T-cell, CD8+ T-cell, and T-cell exhaustion gene signature expression in pre-infusion tumor tissue across the patients. 12](#_Toc87612727)

[REFERENCES 13](#_Toc87612728)

# SUPPLEMENTARY METHODS

## HLA and MAGE-A10 Testing

Patients had to be positive for human leukocyte antigen (HLA)-A*02:01 and/or HLA-A*02:06 alleles and negative for HLA*02:05, HLA-B*15:01, and/or HLA-B*46:01, as determined by central testing at the Histocompatibility and Molecular Genetics Laboratory, American Red Cross (Philadelphia, Pennsylvania). Exclusions for HLA-A*02:05, B*15:01 and B*46:01 were implemented due to potential T-cell receptor (TCR) alloreactivity concerns raised during preclinical evaluation of the TCR (Adaptimmune, data not shown). In addition, tumor samples (either an archival specimen or a fresh biopsy) were pathologically reviewed, and melanoma-associated antigen (MAGE)-A10 immunohistochemical (IHC) staining was performed by QualTek Molecular Laboratories (Goleta, California) using an affinity-purified goat polyclonal antibody (MAGE-A10 (S-14): sc-324906; Santa Cruz Biotechnology, Santa Cruz, California) in a Clinical Laboratory Improvement Amendments- (CLIA-) validated assay.

Using a MAGE-A10 clinical trial assay (analytically validated and certified by the CLIA), IHC positivity was determined by a pathologist based on both percentage of positive tumor cells and intensity of expression, as determined by percent score of IHC staining. All treated patients’ tumor biopsies had ≥30% of tumor cells that were ≥2+ and/or 3+ intensity by IHC staining except patient 4 (dose group 3), whose percent score was 25%. Tumor MAGE-A10 expression for eligibility was derived by calculating P-score and presented for translational correlative studies using Histoscore (H-score). Tumor biopsies of all treated patients met the expression requirements at the time of screening. The H-score was derived from the P-score by 1 × (% of 1+ cells) + 2 × (% of 2+ cells) + 3 × (% of 3+ cells). HLA screening and MAGE-A10 IHC testing was performed according to the protocol, ADP-0000-001 – A Screening Protocol to Determine Tumor Antigen Expression and HLA Sub-Type for Eligibility Determination for Clinical Trials Evaluating the Safety and Efficacy of Autologous T-Cells Expressing Enhanced TCRs in Subjects with Solid or Hematological Malignancies (<https://clinicaltrials.gov>: NCT02636855).

## T-cell Manufacturing

ADP-A2M10 was manufactured and expanded using Xuri™ Cell Expansion System (Cytiva Life Sciences, Marlborough, Massachusetts) at Minaris Regenerative Medicine, LLC (formerly Hitachi Chemical Advanced Therapeutics Solutions, LLC, Allendale, New Jersey) and Adaptimmune, LLC (Philadelphia, Pennsylvania) using vectors manufactured and supplied by City of Hope (Duarte, California), Cincinnati Children’s Hospital Medical Center (Cincinnati, Ohio), or Lentigen Technology Inc. (Gaithersburg, Maryland). The vector was developed to encode α and β TCR sequences separated by a 2A skip sequence driven by an elongation factor-1α promoter. Final cell product release was contingent on several different specifications, including CD3+ ≥80% of cells, ≥10% transduced cells, vector copy number ≤5.0, and cell viability ≥70%; the final ADP-A2M10 product was cryopreserved in 5% dimethyl sulfoxide and thawed prior to intravenous administration.

## Gene Therapy-Related Delayed Adverse Events, Molecular Replication Competent Lentivirus Testing, and Insertional Oncogenesis

Gene therapy-related delayed adverse events (AEs) included new malignancies, new incidence or exacerbation of a pre-existing neurological disorder, new incidence or exacerbation of a prior rheumatologic or other autoimmune disorder, new hematologic disorder, opportunistic or serious infections, or unanticipated illness and/or hospitalization deemed related to gene modified cell therapy. The gene therapy-related delayed AEs, molecular replication competent lentivirus testing (quantitative PCR for the vesicular stomatitis virus-G DNA sequence) and, if necessary, insertional oncogenesis were assessed at 3, 6, and 12 months during year 1; then every 6 months from year 2–5; then annually up to year 15.

## Translational

### Transcriptomic Analysis

Transcriptomic analyses were performed as previously described (1) with the following modifications. Background gene count levels were determined for each sample using mean plus two standard deviations of the included negative control counts. For data normalization, background-subtracted counts were then scaled using geometric means of 27 housekeeping genes shared between the two gene panels. The following gene signatures were used: antigen processing machinery (2), CD3+ T-cells, CD8+ T-cells (3) (NanoString Technologies, Seattle, Washington), and T-cell exhaustion (4). The heatmap represents scores standardized per gene. The scores were generated as number of standard deviations away from the mean (z-score).

### RNA In Situ Hybridization

RNA *in situ* hybridization (RNAish) for the ADP-A2M10 TCR was performed using the RNAscope 2.5 LS Red kit (Advanced Cell Diagnostics, Newark, California) and RNAscope probes specific to ADP-A2M10 TCR (Advanced Cell Diagnostics, Newark, California) according to the manufacturer’s instructions. Briefly, 4-μm formalin-fixed, paraffin-embedded tissue sections were pre-treated with heat and protease prior to hybridization with the target oligo probes. Pre-amplifier, amplifier, and AP-labeled oligos were then hybridized sequentially. RNAish assay was followed by CD3 chromogenic precipitate IHC (anti-CD3 (2GV6) monoclonal primary antibody (Roche Diagnostics, Indianapolis, Indiana)) using the DISCOVERY Teal HRP detection kit (Roche Diagnostics). Each sample was quality-controlled for RNA integrity with a RNAscope probe specific to peptidylprolyl isomerase B (PPIB) RNA (Hs-PPIB, Advanced Cell Diagnostics, Newark, CA). A specific RNA staining signal was identified as red punctate dots and CD3 by the Teal signal. Samples were counterstained with hematoxylin. The whole slides were scanned using AxioScan.Z1 microscope slide scanner and were analyzed using HALO image analysis software (Indica Labs, Albuquerque, New Mexico). Images were annotated and analyzed using Indica Labs - ISH v3.3.9 algorithm with CD3 staining as the nuclear stain, which resulted in CD3+ cell count and ADP-A2M10 TCR percentage positivity.

# SUPPLEMENTARY RESULTS

## Patient Characteristics

A total of 979 patients with head and neck squamous cell carcinoma (HNSCC; 366), melanoma (447), or urothelial carcinoma (UC; 166) signed informed consent and were enrolled in ADP 0000-001 for HLA and tumor antigen testing to determine eligibility for enrollment in Adaptimmune clinical studies. ADP-0000-001 is an umbrella screening protocol developed for multiple TCRs and multiple clinical protocols (i.e., ADP-0022-004, ADP-0022-003, and other studies). HLA alleles were assessed for ADP-A2M10 and other TCRs under this screening protocol. As a result, patients had HLA alleles assessed for all clinical studies though they may not have been screened for an ADP-A2M10 study, but rather, for another study and vice versa. There were 442 patients, including patients with HNSCC (161), melanoma (201), and UC (80) who had HLA alleles that matched the eligibility criteria for ADP‑A2M10. Of those, 323 patients with HNSCC (129), melanoma (143), and UC (51) were screened for the MAGE-A10 antigen (**Supplementary Figure 1**). There were 64 patients with HNSCC (12), melanoma (35), and UC (17) who were ultimately eligible for enrollment (**Supplementary Table 1**). Eighteen patients with appropriate HLA phenotype and tumor MAGE-A10 expression, and who met all other eligibility requirements, signed informed consent for the study, and underwent leukaphereses. From September 2017 to September 2019, 10 patients underwent lymphodepletion and were treated with ADP-A2M10. Of the eight patients who did not receive lymphodepletion chemotherapy or ADP-A2M10, eligibility criteria were not met prior to lymphodepletion in three patients, ADP-A2M10 was unable to be manufactured for one patient, and the study ended prior to treatment for four patients.

# SUPPLEMENTARY TABLES

## Supplementary Table 1. ADP-0022-004 patients screened for HLA and MAGE-A10 under screening protocol ADP-0000-001.

| **Tumor site** | **Number of patients** | | | | | | |
| --- | --- | --- | --- | --- | --- | --- | --- |
|  | **HLA screened under screening protocol for multiple studies^a^** | **Patients with HLA alleles matching ADP-A2M10 eligibility criteria** | **MAGE-A10 antigen screened** | **MAGE-A10 antigen positive^b^** | **MAGE-A10 antigen negative^b^** | **MAGE-A10 antigen not evaluable^c^** | **Dosed with ADP-A2M10 in ADP-0022-004** |
| Head and neck | 366 | 161 | 129 | 12 | 108 | 9 | 4 |
| Melanoma | 447 | 201 | 143 | 35 | 99 | 9 | 3 |
| Bladder | 166 | 80 | 51 | 17 | 29 | 5 | 3 |
| Total | 979 | 442 | 323 | 64 | 236 | 23 | 10 |

^a^ADP-0000-001 (NCT02636855) screened patients for ADP-0022-004 and other studies throughout the time period of this trial.

^b^MAGE-A10 positive and negative staining by immunohistochemistry was determined by the cut-off at the time of testing.

^c^Not evaluable defined as: tumor content insufficient (i.e., <100 tumor cells, no tumor present, ineligible sample type, or ineligible cancer indication/histology).

HLA, human leukocyte antigen; MAGE, melanoma-associated antigen.

## Supplementary Table 2. All AEs related^a^ to ADP-A2M10 by grade ≥3 (mITT population).

|  | **Number of patients (N = 10)** | | |
| --- | --- | --- | --- |
| **Preferred term** | **Any grade** | **Grade 3** | **Grade 4** |
| Patients with any AE | 9 | 4 | 4 |
| Pyrexia | 5 | 1 | 0 |
| Rash | 4 | 0 | 1 |
| Neutropenia/neutrophil count decreased | 3 | 1 | 1 |
| Anemia/red blood cell count decreased | 2 | 2 | 0 |
| CRS | 2 | 1 | 0 |
| Thrombocytopenia/platelet count decreased | 2 | 0 | 1 |
| Alanine aminotransferase increased | 1 | 1 | 0 |
| Amylase increased | 1 | 0 | 1 |
| Aspartate aminotransferase increased | 1 | 0 | 1 |
| Hypermagnesemia | 1 | 1 | 0 |
| Hypotension | 1 | 1 | 0 |
| Leukopenia/white blood cell count decreased | 1 | 0 | 1 |
| Lymphopenia/lymphocyte count decreased | 1 | 1 | 0 |
| Pancytopenia | 1 | 1 | 0 |

^a^Related AEs include definitely, probably, or possibly related events.

AE, adverse event; CRS, cytokine release syndrome; mITT, modified intent-to-treat.

## Supplementary Table 3. Peak persistence and time to peak persistence by dose group (mITT population).

|  | **Group 1**  **(n = 3)** | **Group 3**  **(n = 3)** | **Expansion group**  **(n = 4)** | **Overall**  **(N = 10)** |
| --- | --- | --- | --- | --- |
| Peak persistence (copies/microgram of DNA) | | | | |
| Mean | 3397.9 | 97,827.5 | 70,898.6 | 58,727.1 |
| Median | 1264.9 | 82,548.9 | 64,160.1 | 55,642.5 |
| Range | 193.9–8734.9 | 76,591.6–134,342.0 | 13,270.1–142,004.2 | 193.9–142,004.2 |
| Time to peak persistence (days) | | | | |
| Mean | 8.7 | 3.3 | 3.5 | 5.0 |
| Median | 11.0 | 4.0 | 2.0 | 4.0 |
| Range | 4–11 | 2–4 | 2–8 | 2–11 |

mITT, modified intent-to-treat.

# SUPPLEMENTARY FIGURES

~~
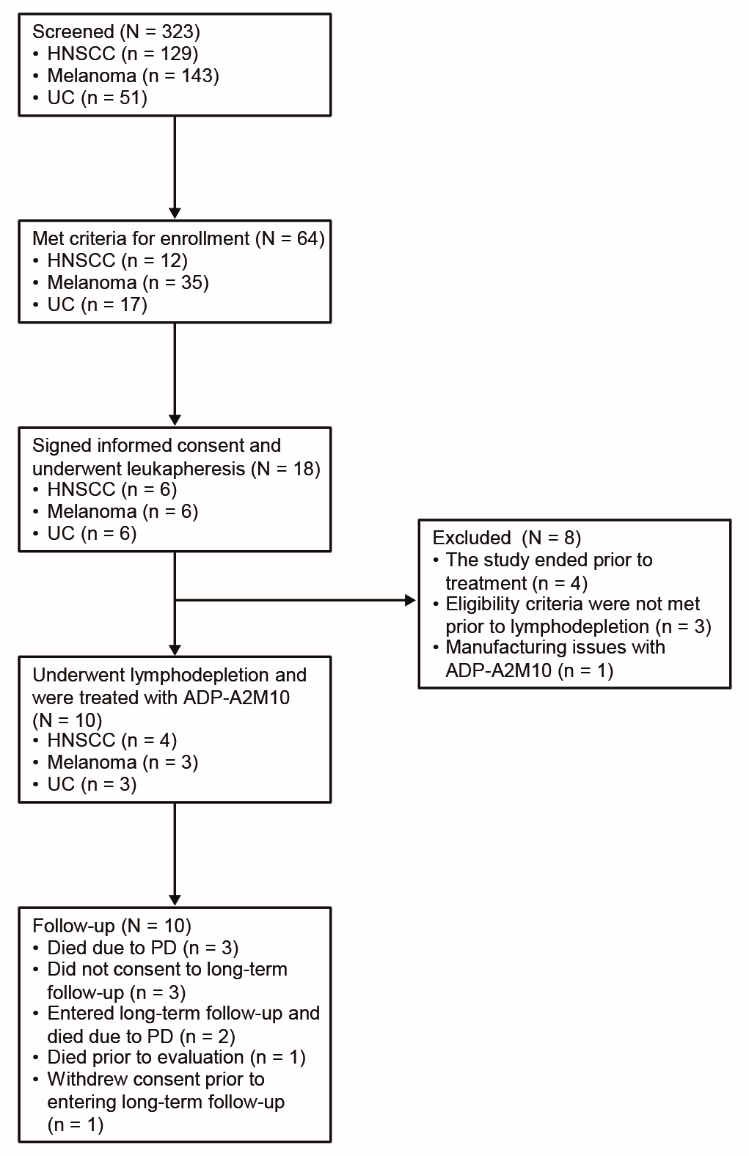
~~

## Supplementary Figure 1. Patient disposition.

HNSCC, head and neck squamous cell carcinoma; PD, progressive disease; UC, urothelial carcinoma.


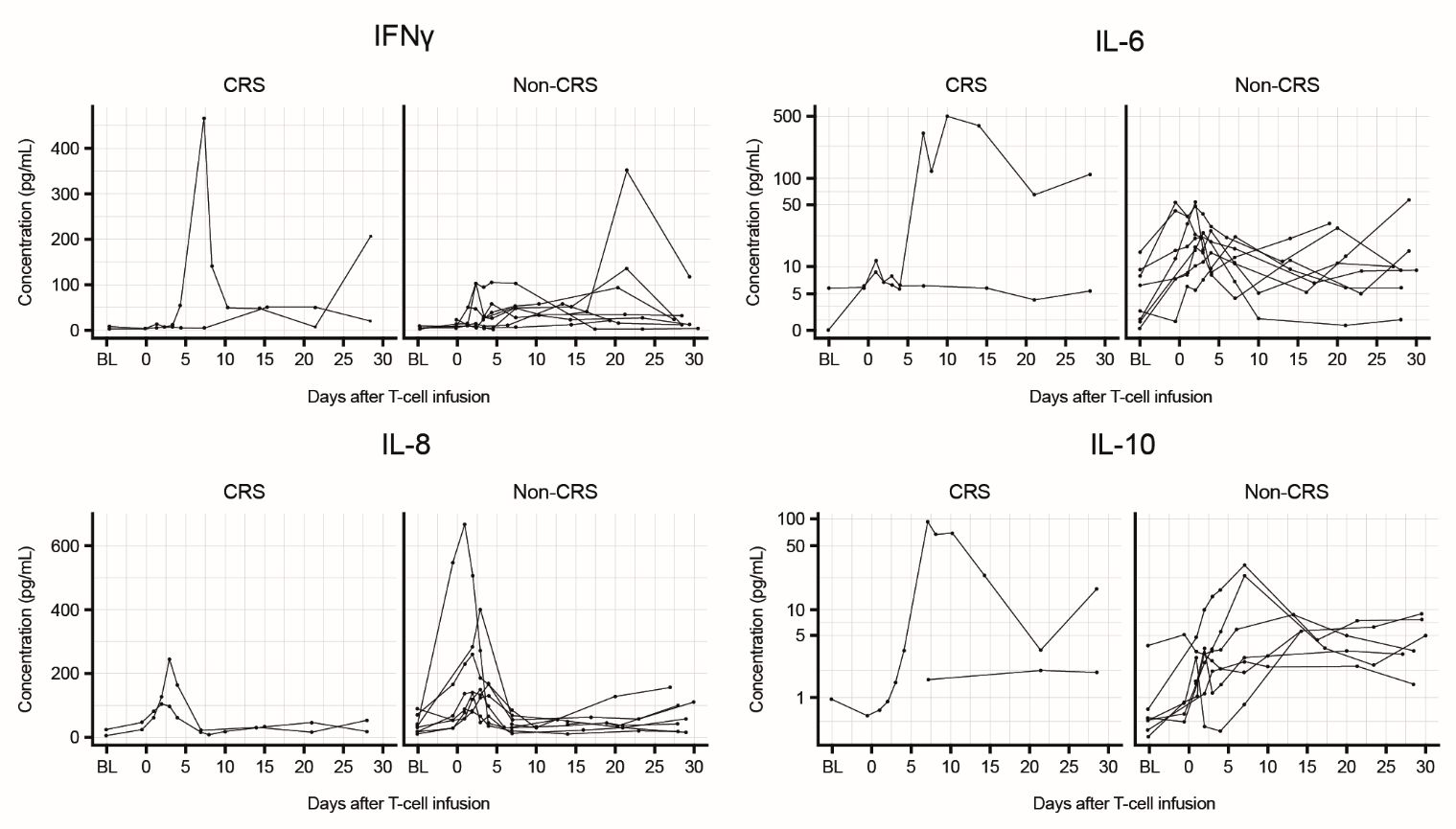


## Supplementary Figure 2. Elevation of selected serum cytokines after infusion in mITT population.

Serum IFNγ, IL-6, IL-8, and IL-10 were measured via a multiplexed electrochemiluminescence-based immunoassay (Meso Scale Diagnostics, LLC, Rockville, Maryland) at the indicated days pre- and post-infusion. Patients with (left panels) and without (right panels) CRS events are shown separately. BL, baseline; CRS, cytokine release syndrome; IFN, interferon; IL, interleukin; mITT, modified intent-to-treat.


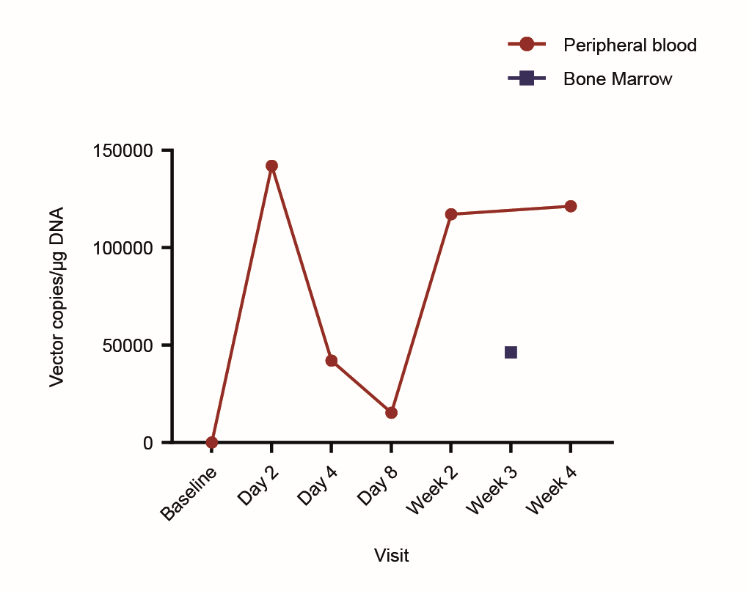


## Supplementary Figure 3. There was no evidence of enrichment of ADP-A2M10 in bone marrow of patient 10 compared with peripheral blood.

Persistence of ADP-A2M10 was measured by quantitative PCR of the Psi element sequence in genomic DNA extracted from peripheral blood and bone marrow mononuclear cells.


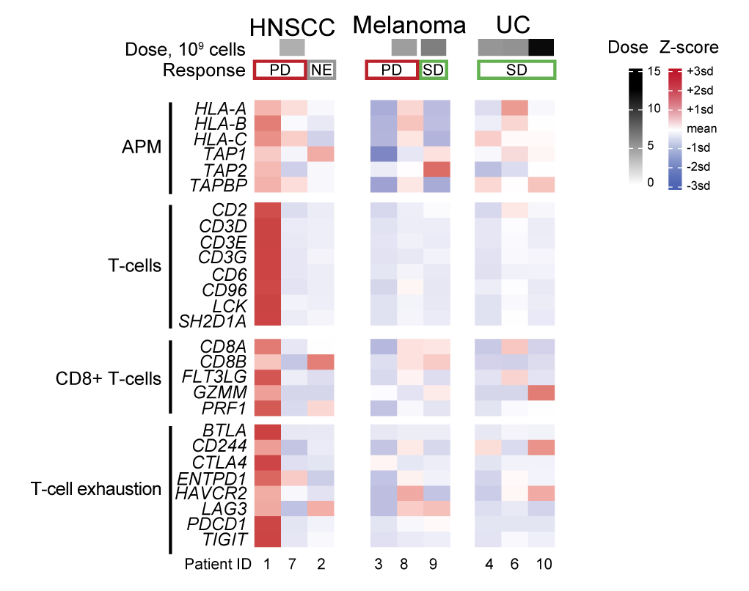


## Supplementary Figure 4. Variability of APM, CD3+ T-cell, CD8+ T-cell, and T-cell exhaustion gene signature expression in pre-infusion tumor tissue across the patients.

Gene expression analysis was performed using the NanoString nCounter system (NanoString Technologies, Seattle, Washington) and presented as a heatmap of scores standardized per gene. The scores were obtained as number of standard deviations away from the mean (z-score). Patient 5 did not have enough tumor for assessment. APM, antigen processing machinery; HNSCC, head and neck squamous cell carcinoma; NE, not evaluable; PD, progressive disease; SD, stable disease; UC, urothelial carcinoma.

# REFERENCES

1. Ramachandran I, Lowther DE, Dryer-Minnerly R, Wang R, Fayngerts S, Nunez D, et al. Systemic and Local Immunity Following Adoptive Transfer of NY-ESO-1 SPEAR T Cells in Synovial Sarcoma. *J Immunother Cancer* (2019) 7(1):276. doi: 10.1186/s40425-019-0762-2

2. Şenbabaoğlu Y, Gejman RS, Winer AG, Liu M, Van Allen EM, de Velasco G, et al. Tumor Immune Microenvironment Characterization in Clear Cell Renal Cell Carcinoma Identifies Prognostic and Immunotherapeutically Relevant Messenger RNA Signatures. *Genome Biol* (2016) 17(1):231. doi: 10.1186/s13059-016-1092-z

3. Rhodes M, Danaher P, Dennis L, Mashadi-Hossein A, Irving L, Beechem J. *Using the PanCancer Immune Profiling Advanced Analysis Module* (2019). Available at: https://www.nanostring.com/wp-content/uploads/2020/12/WP_MK1190_PanCancer_Immune_Profiling_Advanced_Analysis.pdf.

4. Woroniecka K, Chongsathidkiet P, Rhodin K, Kemeny H, Dechant C, Farber SH, et al. T-Cell Exhaustion Signatures Vary With Tumor Type and are Severe in Glioblastoma. *Clin Cancer Res* (2018) 24(17):4175–86. doi: 10.1158/1078-0432.CCR-17-1846
